# Molecular Detection of Tick-Borne Pathogens and Genetic Diversity of *Theileria orientalis* in Swamp Buffaloes from Northeast Thailand

**DOI:** 10.3390/ani16121876

**Published:** 2026-06-17

**Authors:** Supawadee Piratae, Tossapol Seerintra, Wasupon Chatan, Kotchaphon Vaisusuk

**Affiliations:** 1 Faculty of Veterinary Sciences, Mahasarakham University, Maha Sarakham 44000, Thailand; supawadee.p@msu.ac.th (S.P.); tossapol.s@msu.ac.th (T.S.); wasupon.c@msu.ac.th (W.C.); 2 One Health Research Unit, Mahasarakham University, Maha Sarakham 44000, Thailand; 3 Veterinary Infectious Disease Research Unit, Mahasarakham University, Maha Sarakham 44000, Thailand; 4 Department of Veterinary Technology and Veterinary Nursing, Faculty of Agricultural Technology, Rajabhat Maha Sarakham University, Maha Sarakham 44000, Thailand

**Keywords:** genetic diversity, northeastern Thailand, piroplasmida, swamp buffaloes, tick borne pathogens

## Abstract

Blood-borne parasites, including *Babesia* spp., *Theileria* spp., and *Anaplasma* spp., pose significant threats to buffalo health and productivity, highlighting the need for continuous epidemiological surveillance in endemic regions such as Thailand. This study aimed to investigate the molecular prevalence of hemoparasites and characterize the genetic diversity of *Theileria orientalis* in swamp buffaloes from Northeast Thailand. A total of 176 blood samples were analyzed using genus- and species-specific PCR assays. Samples positive for *T. orientalis* were subjected to DNA sequencing of the *MPSP* gene, followed by haplotype and phylogenetic analyses. Overall, 19.3% (95% CI: 13.8–25.9) of samples were positive for at least one hemoparasite. The prevalence of *T. orientalis*, *Babesia bigemina*, and *B. bovis* was 15.9%, 3.4%, and 2.3%, respectively. Notably, all *B. bovis* infections occurred as co-infections with *T. orientalis*. *Anaplasma* spp. showed the lowest prevalence (1.7%), comprising *A. marginale* 0.6% and *A. platys* 1.1%. Sequence analysis of *T. orientalis* revealed significant genetic diversity within the population. Subsequent phylogenetic and pairwise distance analyses identified four distinct genotypes including Type 7, which was found to be predominant, followed by Type 5, Type N2 and Type 4. This study provides molecular evidence of hemoparasite infections and highlights the complex genetic diversity of *T. orientalis* in Thai water buffaloes. The co-circulation of multiple genotypes and co-infections underscore the need for improved epidemiological monitoring and targeted control strategies in the region.

## Simple Summary

Blood-borne parasites pose a significant health concern for swamp buffaloes, reducing productivity and causing economic losses in livestock systems. In this study, we examined buffaloes in northeastern Thailand and found that approximately 19.3% of animals were infected with at least one parasite. *Theileria orientalis* was identified as the most prevalent species, while other parasites, including *Babesia bigemina*, *Babesia bovis*, and *Anaplasma* spp., were detected at lower frequencies. Co-infections were also observed, which may increase disease severity. Further genetic analysis of *T. orientalis* revealed substantial diversity, with four genotypes identified. Type 7 was the most prevalent, while Types 5, N2 and 4 were also present. These genotypes showed close genetic relationships to strains reported in other countries, suggesting widespread distribution of similar parasite populations across regions. Overall, these findings underscore the need for continued surveillance and improved control strategies to safeguard buffalo health and sustain livestock productivity.

## 1. Introduction

Livestock production is a vital global economic activity, contributing significantly to agricultural Gross Domestic Product (GDP), rural livelihoods, and food security, particularly in tropical and developing regions such as Thailand [1]. In the country’s northeastern region, swamp buffaloes (*Bubalus bubalis*) are extensively raised and play a crucial role in agricultural systems. Beyond their local importance, buffaloes are globally significant for their contributions to sustainable farming, draft power, and nutrient-rich food production, thereby supporting rural economies and enhancing resilience in smallholder systems [2]. Despite their economic and cultural importance, buffalo productivity is significantly constrained by hemoparasitic infections, notably those caused by *Babesia* spp., *Theileria* spp., and *Anaplasma* spp. These pathogens cause substantial morbidity and mortality, leading to reduced growth performance, decreased work capacity, and increased treatment costs, thereby imposing considerable economic losses on smallholder farmers [3,4].

Protozoan parasites of the genera *Babesia* and *Theileria* (phylum Apicomplexa) are the causative agents of babesiosis and theileriosis, respectively. In Thailand, *B. bovis* and *B. bigemina* have been widely reported in swamp buffaloes [5], while infections with *T. orientalis* have also been documented [4,6]. In addition to protozoan pathogens, bacterial hemoparasites such as *A. marginale* [7] and *A. platys* [8] have been detected in buffalo populations.

*Babesia bovis* and *B. bigemina* are primarily transmitted by the Asian blue tick, *Rhipicephalus microplus* [9], whereas *T. orientalis* is mainly associated with ticks of the genus *Haemaphysalis* [10]. *Babesia* spp. are intraerythrocytic parasites that cause babesiosis, which may present with anemia, jaundice, hemoglobinuria, and, in severe cases, death. Infections with *B. bovis* are frequently associated with neurological signs due to sequestration of parasitized erythrocytes in cerebral capillaries [9,11]. In contrast, *Theileria* spp. infect both erythrocytes and leukocytes and may induce lymphoproliferative disorders, immunosuppression, and chronic anemia, leading to reduced productivity [12]. Moreover, oriental bovine theileriosis caused by *T. orientalis* is widely distributed in Asia and has been linked not only to *Haemaphysalis* ticks but also to *R. microplus*, *Dermacentor* spp., and mechanical transmission by tabanid flies [10].

*Anaplasma* spp. are obligate intracellular, Gram-negative bacteria belonging to the family Anaplasmataceae. Among them, *A. marginale* is the principal etiological agent of anaplasmosis in buffaloes, infecting erythrocytes and causing progressive hemolytic anemia, fever, weight loss, reproductive failure, and decreased milk production [13]. Transmission of *Anaplasma* spp. occurs mainly via ixodid ticks, particularly *Rhipicephalus* spp., although mechanical transmission through biting flies and iatrogenic routes has also been documented [14]. Persistently infected carrier animals play a critical role as reservoirs, facilitating long-term pathogen maintenance and dissemination within herds [14].

Accurate diagnosis is essential for effective disease management. While conventional microscopy and serology are commonly used in field settings due to their low cost, they lack the sensitivity to detect low parasitemia and have limited ability to differentiate species in mixed infections. Molecular techniques, particularly polymerase chain reaction (PCR), offer superior sensitivity and enable specific species identification. Genetic markers such as the spherical body protein 2 (*sbp-2*) gene for *B. bovis* [15], the rhoptry-associated protein 1a (*rap-1a*) gene for *B. bigemina* [16], the major piroplasm surface protein (*MPSP*) gene for *Theileria* spp. [17], the major surface protein 4 (*msp4*) gene for *A. marginale*, and the 16S rRNA gene for *A. platys* are widely used for molecular detection and species differentiation [18].

Despite the recognized economic and epidemiological importance of hemoparasitic infections in Thai livestock, comprehensive molecular studies focusing on water buffaloes, particularly in northeastern Thailand, remain limited. Therefore, this study aimed to investigate the prevalence and species composition of *Babesia* spp., *Theileria* spp., and *Anaplasma* spp., as well as the molecular diversity of *Theileria* spp., in swamp buffaloes from northeastern Thailand. The findings are expected to enhance understanding of hemoparasite epidemiology and support the development of effective surveillance, prevention, and control strategies for these infections in buffalo populations.

## 2. Materials and Methods

### 2.1. Study Area and Sample Collection

**Figure 1:**
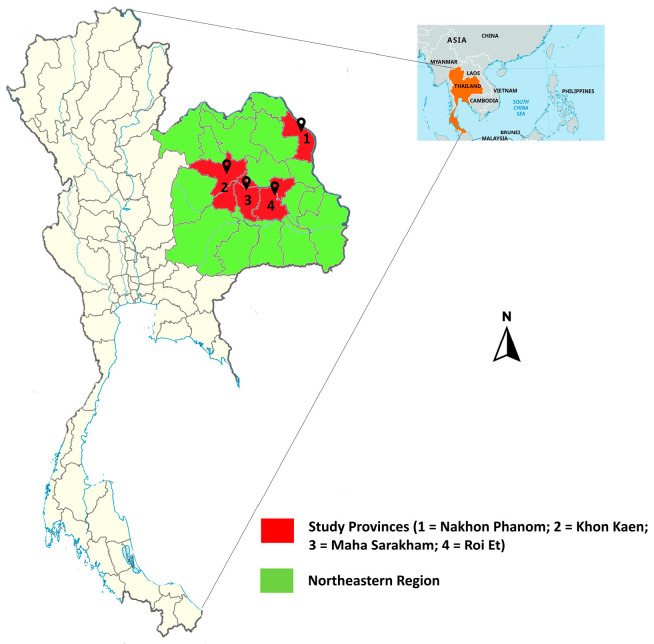
Map of the study area, showing four provinces in the northeastern part of Thailand where the blood samples were collected.

The study was conducted in four provinces in northeastern Thailand, including Khon Kaen (n = 20), Maha Sarakham (n = 84), Roi Et (n = 42), and Nakhon Phanom (n = 30) (Figure 1), a region characterized by a tropical savanna climate and mixed livestock agriculture systems. A cross-sectional study design was employed to investigate the prevalence of tick-borne pathogens in swamp buffaloes (*B. bubalis*). Sampling was conducted between July and October 2025, during the rainy season, to account for potential seasonal variations in pathogen prevalence. Based on an expected pathogen prevalence of approximately 13% from a previous study [5], 95% confidence level (α = 0.05), and 5% margin of error, the minimum required sample size was calculated to be 174 using Cochran’s formula. Consequently, a total of 176 blood samples were collected from healthy, asymptomatic swamp buffaloes in smallholder farms and community grazing areas. Approximately 3 mL of blood was collected via jugular venipuncture using EDTA-coated vacutainer tubes. Samples were stored on ice during transport to the laboratory and either processed immediately or stored at −20 °C until DNA extraction. All sample collections were conducted with the consent of the animal owners and were approved by the relevant ethics committee. All experimental procedures involving animals were approved by the Institutional Animal Care and Use Committee, Mahasarakham University (IACUC-MSU-038-021/2025).

### 2.2. DNA Extraction and PCR Amplification for Screening of Hemoparasitic Infections

Genomic DNA was extracted from 200 µL of whole blood using the GF-1 Blood DNA Extraction Kit (Vivantis, Kuala Lumpur, Malaysia) in accordance with the manufacturer’s instructions. All DNA samples were stored at −20 °C until further molecular analysis. All extracted DNA samples were initially screened for piroplasm infections (*Babesia* spp. and *Theileria* spp.) using a nested polymerase chain reaction (nested PCR) assay targeting the 18S rRNA gene with BTH primers [19].

To achieve taxonomic resolution and differentiate between the two genera, samples that tested positive in the initial screening were subsequently examined using a *Babesia* genus-specific conventional PCR assay (targeting the 18S rRNA gene via Ba103-F/Ba721-R primers) [20]. Samples confirmed positive for *Babesia* spp. were further characterized using species-specific nested PCR assays. *B. bovis* was detected by targeting the spherical body protein 2 (*sbp-2*) gene [15], and *B. bigemina* was detected by targeting the rhoptry-associated protein 1a (*rap-1a*) gene [21,22]. Concurrently, all piroplasm-positive samples from the initial nested PCR screening were analyzed for *T. orientalis* infections using a PCR assay targeting the major piroplasm surface protein (MPSP) gene [23].

All DNA samples were also screened for the family Anaplasmataceae using a conventional PCR assay targeting the 16S rRNA gene which was selected to capture a broad range of *Anaplasma* species [24]. Samples yielding positive results were further analyzed using species-specific PCR assays to resolve them to the species level. Detection of *A. marginale* was conducted using primers targeting the major surface protein 4 (*msp4*) gene [25]. Additionally, nested PCR assays targeting the 16S rRNA gene were employed for the detection of *A. platys* and *A. bovis* [26,27]. The first amplification round utilized genus-specific outer primers, followed by a second round using species-specific inner primers for each pathogen (Table 1).

All PCRs were carried out in a total volume of 20 μL, comprising 10 μL of 2X PCR i-StarMAX^TM^ II (Intron Biotechnology, Seongnam, Republic of Korea), 1 μL of each primer (10 μM), 5 μL of distilled water (DW), and 3 μL of DNA template. Thermal cycling conditions consisted of an initial denaturation at 94 °C for 3 min, followed by 35 cycles of denaturation at 94 °C for 45 s, annealing at 49–58 °C for 45 s depending on the primer set, and extension at 72 °C for 60 s, with a final extension at 72 °C for 10 min. Positive controls consisted of DNA extracted from cattle confirmed to be infected with *Babesia* spp., *Theileria* spp., and *Anaplasma* spp. from previous study [28,29]. Negative controls included PCR master mixes containing primers but no DNA template. PCR products were visualized by electrophoresis on 1% agarose gels stained with ViSafe Red Gel Stain (Vivantis, Kuala Lumpur, Malaysia) and observed under ultraviolet light using a gel documentation system. Only samples exhibiting bands of the correct expected size were considered positive, in accordance with the amplicon sizes (Table 1).

### 2.3. Sequence and Phylogenetic Analysis of T. orientalis

All *T. orientalis*-positive samples were purified and subjected to sequencing by a commercial service (U2Bio, Seoul, Republic of Korea). The obtained nucleotide sequences were manually trimmed and edited using BioEdit version 7.2 software. Multiple sequence alignments were performed using the ClustalW algorithm implemented in BioEdit [30]. Sequence similarity was assessed using the Basic Local Alignment Search Tool (BLAST implemented within BioEdit version 7.2) against reference sequences available in the National Center for Biotechnology Information (NCBI) database at https://www.ncbi.nlm.nih.gov/ (accessed on 11 June 2026). Haplotype diversity and nucleotide variation were analyzed using DnaSP version 6 [31]. Representative sequences generated in this study were deposited in the GenBank database. Pairwise genetic distances and phylogenetic relationships were inferred using MEGA 11 software [32]. The phylogenetic tree was reconstructed based on the Maximum Likelihood (ML) method using the Tamura–Nei model. The robustness of the inferred phylogenetic trees was evaluated using 1000 bootstrap replicates. Reference sequences retrieved from GenBank were included for comparative analysis.

### 2.4. Statistical Analysis

The prevalence of each hemoparasite species was calculated using descriptive statistical analysis. Infection rates were expressed as percentages based on the number of positive samples relative to the total number of samples examined. Confidence intervals (95% CI) were calculated to assess the precision of prevalence estimates and to facilitate comparisons among parasite species. The occurrence of mixed infections was also recorded and analyzed accordingly.

## 3. Results

### 3.1. Prevalence of Hemoparasites in Water Buffaloes

**Figure 2:**
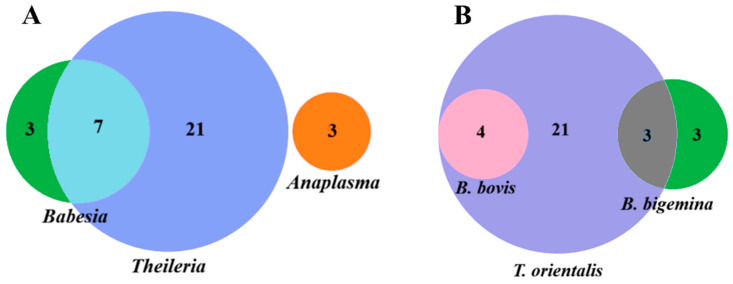
Diagram of the infection patterns. (**A**): Hemoparasite infections in Thai buffaloes; (**B**): Co-infection of piroplasmid parasites in Thai buffaloes.

Overall, PCR screening revealed that 19.3% (34/176) of samples were positive for at least one hemoparasite, while 80.7% (142/176) were negative. When analyzed by region, Nakhon Phanom exhibited the highest prevalence at 36.7%, followed by Maha Sarakham at 19.1%, Roi Et at 11.9% and Khon Kaen at 10.0%. *Theileria orientalis* was the predominant pathogen detected (15.9%). *Babesia* infections occurred at lower frequencies, with *B. bigemina* (3.4%) and *B. bovis* (2.3%) identified. *Anaplasma* spp. exhibited the lowest prevalence (1.7%), with detected species including *A. marginale* and *A. platys*, while *A. bovis* was not identified (Table 2). Co-infections involving two parasite species were identified in seven samples (4.0%). The most common co-infection pattern was *B. bovis* with *T. orientalis* (4/176), followed by *B. bigemina* with *T. orientalis* (3/176) (Figure 2). No triple infections involving all three genera were detected.

### 3.2. Sequencing Analysis and Genetic Diversity of Theileria orientalis

A total of 28 *T. orientalis*-positive samples were subjected to sequencing of the partial *MPSP* gene, with 27 yielding high-quality sequences suitable for analysis. Haplotype analysis identified 15 distinct haplotypes, with a high level of genetic diversity within the studied population (Hd = 0.968, π = 0.11072). The representative sequences for each of these 15 distinct haplotypes have been deposited in GenBank under accession numbers PZ316040–PZ316054 (Table 3). Genotype classification revealed the presence of four *T. orientalis* genotypes. Type 7 was the predominant genotype, followed by Type 5, Type N2 and Type 4, respectively. Sequence similarity analysis by BLAST showed that all haplotypes exhibited high intra-type nucleotide identity (99.3–100%) with previously reported *T. orientalis* sequences in the NCBI GenBank database from diverse geographical regions, suggesting widespread distribution and genetic relatedness of these genotypes.

Pairwise genetic relationships among the 15 haplotypes (Table 4) revealed low genetic divergence within genotypes (0.00143–0.03611) and substantially higher divergence between genotypes (0.16876–0.26777). Consistently, the mean pairwise genetic distance analysis (Table 5) demonstrated substantial inter-genotypic divergence, ranging from 0.170 to 0.261, with the greatest distance observed between Type 5 and Type N2 (0.261), whereas the lowest was detected between Type 4 and Type 5 (0.170). In contrast, intra-genotypic distances were markedly lower, with mean values of 0.020 for both Type 7 and Type N2, and 0.01 for Type 5.

### 3.3. Phylogenetic Analysis of Theileria orientalis

**Figure 3:**
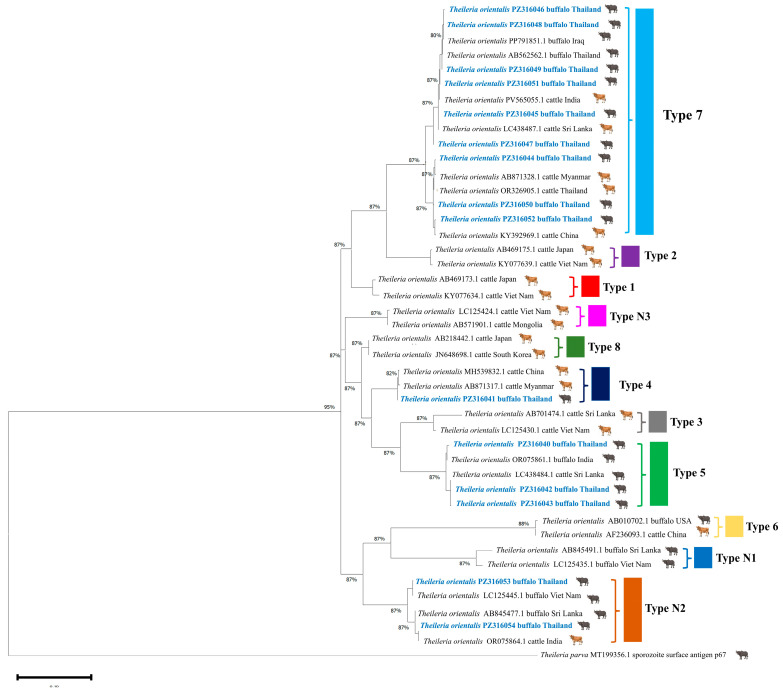
Phylogenetic tree based on partial *MPSP* gene sequences of 15 *T. orientalis* haplotypes identified in this study (shown in blue bold), along with 28 *T. orientalis* sequences from various geographic regions obtained from the GenBank database. *T. parva* sequence was included as an outgroup. Bootstrap support values (based on 1000 replicates) are shown at the nodes, indicating the percentage of trees in which the associated taxa clustered together. Bootstrap values (≥80%) from 1000 replicates are shown at the nodes.

Phylogenetic analysis based on partial *MPSP* gene sequences confirmed that all isolates obtained in the present study belonged to the *T. orientalis* complex. The phylogenetic tree, constructed using reference sequences representing 11 recognized MPSP genotypes (Types 1–8 and N1–N3), revealed the presence of four genotypes among the examined buffalo isolates: Types 4, 5, 7, and N2 (Figure 3). Type 7 was the predominant genotype and formed a well-supported clade with isolates previously reported from Thailand, India, Sri Lanka, Myanmar, China, and Iraq. In the phylogenetic tree, Type 7 was recovered as a sister lineage to Type 2 (Ikeda). Type 5 formed a well-supported lineage comprising buffalo isolates from India and Sri Lanka and was recovered as a sister group to Type 3 (Buffeli). Similarly, Type N2 clustered with reference sequences from India, Vietnam, and Sri Lanka. The Type 4 isolates clustered with previously reported Type 4 sequences from cattle in China and Myanmar.

## 4. Discussion

Studying hemoparasites in swamp buffaloes, which serve as important natural reservoirs for these pathogens, provides critical insights into parasite diversity and transmission dynamics. In the present study, PCR-based detection revealed an overall hemoparasite prevalence of 19.3% among swamp buffaloes in northeastern Thailand. Notably, *T. orientalis* was detected in 90.3% of infected animals, confirming its dominance within the infected population. Moreover, all animals infected with *B. bovis* were co-infected with *T. orientalis*, indicating the co-occurrence of these pathogens in the examined buffalo population. The occurrence of *Theileria* and *Babesia* co-infections may reflect exposure to similar ecological conditions or transmission pathways [33]. In addition, clinical signs associated with *T. orientalis* infection may compromise host immunity, thereby increasing susceptibility to secondary infections with more pathogenic species such as *B. bovis* [34]. Furthermore, the overall co-infection rate of 22.6% among positive samples highlights that multi-parasitism is relatively common in buffalo populations, which may complicate clinical diagnosis and negatively affect animal productivity. However, the present study did not investigate vector populations, pathogen interactions, or host immune responses; therefore, the mechanisms underlying this co-occurrence remain unclear and warrant further investigation.

Compared with previous studies, relatively few investigations have specifically examined hemoparasite prevalence in buffaloes in Thailand, as most research has focused on cattle. This study reports parasite prevalence in comparison with previously published data. PCR-based studies have identified piroplasm prevalence rates of approximately 25.4% in buffaloes [35]. Infections with *B. bovis* (11.2%) and *B. bigemina* (3.6%) have also been documented in swamp buffaloes [5], while other studies recently have reported lower prevalence rates of 2.58% and 5.80%, respectively [36]. Similarly, *T. orientalis* infections have been observed across various regions, with reported prevalence as high as 29.37% [4]. In addition, bacterial hemoparasites such as *A. marginale* [7] and *Anaplasma* spp. (6–67.6%) have also been detected, although *A. platys* appear to occur at relatively low prevalence (0.66%) [35]. In contrast, the present study demonstrates a notably low prevalence of *Anaplasma* spp. in buffaloes. This finding is consistent with previous reports showing no positive cases of *A. marginale* in buffaloes from Thailand [37]. These results suggest that buffaloes may be less susceptible to *Anaplasma* infection or may play a limited role as reservoirs compared to cattle. The prevalence variations observed between this study and previous reports can be attributed to several interrelated factors. Beyond differences in sample sizes, diagnostic approaches, and animal management practices, host susceptibility and seasonal variations also influence infection risk. Furthermore, vector-related factors, such as tick abundance and distribution, play a key role in transmission dynamics [38]. These dynamics are heavily shaped by environmental variables, including climate, habitat characteristics, and land-use changes, all of which drive vector ecology and disease epidemiology.

*T. orientalis* exhibits a higher prevalence than *Babesia* spp. in buffaloes, reflecting its endemicity, likely driven by the prevalence of *Rhipicephalus* and *Haemaphysalis* vectors. While *T. orientalis* is often associated with *Haemaphysalis* globally and *B. bovis* and *B. bigemina* are vectored mainly by *R*. *microplus* [9,10], recent evidence in Thailand shows high detection rates in the dominant tick *R. microplus*, which facilitates high co-infection risk alongside *B. bovis* and *B. bigemina* [39]. Furthermore, under communal grazing in northeastern Thailand, buffaloes share pastures with diverse tick species, increasing their risk of acquiring multiple tick-borne pathogens. Additionally, mechanical transmission by hematophagous flies may facilitate pathogen spread [40]. Together, these factors likely explain the observed co-infection patterns.

Haplotype analysis revealed high genetic diversity within the regional *T. orientalis* population, characterized by multiple distinct haplotypes. This diversity is primarily driven by the co-circulation of four genotypes including Type 7, Type 5, Type 4 and Type N2. Pairwise distance analysis confirms that these genotypes represent discrete evolutionary lineages. Specifically, the mean inter-genotypic distances significantly exceed standard divergence thresholds, while the minimal intra-genotypic variation indicates strong genetic conservation within each lineage. Furthermore, our results align with the global characterization which categorized *T. orientalis* into 12 distinct genotypes [41]. The consistency in these diversity profiles and the clear separation of lineages across studies underscore the complex global population structure of *T. orientalis*. Importantly, these findings validate the use of the MPSP gene as a robust marker for lineage differentiation.

In this study, *T. orientalis* Type 7 emerged as the predominant genotype, a finding of significant clinical concern given its increasing association with symptomatic bovine theileriosis [42]. However, its specific impact on the sampled buffaloes could not be assessed because hematological, biochemical, and detailed clinical evaluations were not performed. Future longitudinal studies incorporating clinical tracking and hematological profiling are therefore essential to better define the health impacts and pathogenicity of this genotype in buffalo populations. Phylogenetic reconstruction provided robust support for these clades confirming the reliability of our lineage assignments. Notably, sequences derived from Thai water buffaloes did not form a single monophyletic group but were distributed across multiple clades. This non-monophyletic pattern, characterized by close clustering with reference sequences from cattle and ticks across India, China, Myanmar, Vietnam, Sri Lanka and Thailand, indicates low host specificity and frequent cross-species transmission. Such dynamics are particularly common in mixed farming systems where co-grazing and shared tick vectors facilitate movement between hosts. Furthermore, the clustering of Type 7, 5, 4 and N2 with sequences from Asian countries underscores the epidemiological connectivity within Asia, likely driven by regional livestock trade and transboundary animal movement. A limitation of the present study is that nucleotide sequencing was exclusively performed on *T. orientalis* MPSP-positive amplicons due to their higher prevalence and suitability for phylogenetic characterization. Although the PCR assays for *Babesia* spp. and *Anaplasma* spp. were strictly validated using verified positive controls and demonstrated highly specific amplicon sizes, the absence of sequencing confirmation for these lower-prevalence pathogens remains a limitation regarding definitive genetic identification. Future large-scale surveillance should incorporate sequencing for all PCR-positive samples to definitively characterize the genetic diversity of these tick-borne pathogens in the region.

## 5. Conclusions

In conclusion, this study provides molecular evidence of tick-borne pathogens in asymptomatic swamp buffaloes from northeastern Thailand, with *T. orientalis* identified as the predominant species. Genetic characterization revealed the co-circulation of multiple *T. orientalis* genotypes, highlighting substantial genetic diversity within the studied population. Furthermore, phylogenetic analyses demonstrated close evolutionary relationships between the detected isolates and strains previously reported across Asia, suggesting a regional circulation of genetically related parasites. While the detection of pathogen DNA confirms active infection within the surveyed population, the precise epidemiological role of swamp buffaloes in pathogen maintenance and transmission remains to be fully elucidated. Moreover, the clinical significance of these genotypes, particularly *T. orientalis* Type 7, remains unclear due to the lack of baseline hematological, parasitological, and longitudinal health data. Future research integrating vector surveillance, quantitative PCR-based parasitemia estimation, host health profiling, and livestock movement data is essential to unravel transmission dynamics, evaluate genotype-specific pathogenicity, and inform evidence-based control strategies for tick-borne diseases in Southeast Asia.

## Figures and Tables

**Table 1:** Primers used for PCR amplification of hemoparasites, annealing temperatures, and expected amplicon sizes.

Parasite	Primer Name	Primer Sequence (5′–3′)	Annealing Temp (°C)	Product Size (bp)	Reference
*Babesia* spp./*Theileria* spp.	1st F BTH	GTGAAACTGCGAATGGCTCATTAC	50	~1500	[19]
	1st R BTH	AAGTGATAAGGTTCACAAAACTTCCC			
	2nd F BTH	GGCTCATTACAACAGTTATAGTTTATTTG			
	2nd R BTH	CGGTCCGAATAATTCACCGGAT			
*Babesia* spp.	Ba103-F	CCAATCCTGACACAGGGAGGTAGTGACA	58	619	[20]
	Ba721-R	CCCCAGAACCCAAAGACTTTGATTTCTCTCAAG			
*B. bovis*	Outer F	CTGGAAGTGGATCTCATGCAACC	58 49	1236 580	[15]
	Outer R	TCACGAGCACTCTACGGCTTTGCAG			
	Inner F2	GAATCTAGGCATATAAGGCAT			
	Inner R2	CCCCTCCTAAGGTTGGCTAC			
*B. bigemina*	Outer F	GAGTCTGCCAAATCCTTAC	50 58	879 412	[22] [21]
	Outer R	TCCTCTACAGCTGCTTCG			
	Inner F2	AGCTTGCTTTCACAACTCGCC			
	Inner R2	TTGGTGCTTTGACCGACGACAT			
*T. orientalis*	MPSP-F	CTTTGCCTAGGATACTTCCT	58	776	[23]
	MPSP-R	ACGGCAAGTGGTGAGAACT			
Anaplasmataceae	EHR16SD	GGTACCYACAGAAGAAGTCC	55	345	[24]
	EHR16SR	TAGCACTCATCGTTTACAGC			
*Anaplasma* (outer primer)	EE1	TCCTGGCTCAGAACGAACGCTGGCGGC	55	1430	[26]
	EE2	AGTCACTGACCCAACCTTAAATGGCTG			
*A. platys* (inner primer)	APf	AAGTCGAACGGATTTTTGTC	55	506	[26]
	APr	CTTTAACTTACCGAACC			
*A. bovis* (inner primer)	AB1f	CTCGTAGCTTGCTATGAGAAC	55	551	[27]
	AB1r	TCTCCCGGACTCCAGTCTG			
*A. marginale*	MSP43	GGGAGCTCCTATGAATTACAGAGAATTGTTTAC	56	849	[25]
	MSP45	CCGGATCCTTAGCTGAACAGGAATCTTGC			

**Table 2:** PCR detection results for *Theileria* spp., *Babesia* spp., *Anaplasma* spp., and mixed infections.

Provinces (N)	No. of Single Infection (%)					No. of Mixed Infection (%)		% Prevalence of Hemoparasites (95% CI)
	*A. marginale*	*A. platys*	*B. bovis*	*B. bigemina*	*T. orientalis*	*B. bovis + T. orientalis*	*B. bigemina + T. orientalis*	
Nakhon Phanom (30)	-	-	-	0	7	2	2	36.7 (19.9–56.1)
Maha Sarakham (84)	1	-	-	1	12	2	0	19.1 (11.3–29.1)
Roi Et (42)	-	2	-	1	1	-	1	11.9 (4.0–25.6)
Khon Kaen (20)	-	-	-	1	1	-	0	10.0 (1.2–31.7)
Total (176)	1 (0.6)	2 (1.1)	-	3 (1.7)	21 (11.9)	4 (2.3)	3 (1.7)	19.3 (13.8–25.9)

**Table 3:** Haplotypes of 27 *T. orientalis* sequences from swamp buffaloes in Thailand matched to NCBI GenBank databases.

Haplotype	Sample IDs	*T. orientalis* Genotypes	NCBI GenBank Accession No.	Closest Sequences in NCBI GenBank (% Similarity)
1	RB9, BNP1	Type 4	PZ316041	AB562561.1 (99.8), MH539832.1 (99.8)
2	BKW2, BPK11, BNP6	Type 5	PZ316040	AB560818.1 (99.7), OR075861.1 (99.7)
3	BSP2	Type 5	PZ316042	LC438484.1 (100), AB701450.1 (99.9)
4	BPK15	Type 5	PZ316043	LC438484.1 (99.9), AB701450.1 (99.7)
5	BPK10, BNP3	Type 7	PZ316044	OR326905.1 (99.7), AB871328.1 (99.7)
6	BPK8, BPK7	Type 7	PZ316045	ON457667.1 (100), LC438487.1 (100)
7	BP10, BNP9	Type 7	PZ316046	OR326898.1 (99.6), PP791851.1 (99.6)
8	BNP19	Type 7	PZ316047	ON457667.1 (99.3), LC438487.1 (99.30)
9	BNP16, BKW11, BPK19	Type 7	PZ316048	OR326898.1 (100), PP791851.1 (100)
10	BNP15	Type 7	PZ316049	AB562562.1 (99.9), PP791851.1 (99.7)
11	BNP7, BBB14	Type 7	PZ316050	OM830307.1 (99.9), AB871336.1 (99.9)
12	BNP2, BPK13	Type 7	PZ316051	PV565055.1 (99.9), OP019042.1 (99.9)
13	BKW4	Type 7	PZ316052	KY392969.1 (99.7), OM830307.1 (99.6)
14	BBB6, BPK6, BNP11	Type N2	PZ316053	LC125445 (100), AB560831.1 (99.9)
15	BNP26	Type N2	PZ316054	OR075864.1 (99.6), AB845477.1 (99.6)

**Table 4:** Heatmap of pairwise genetic distances based on the *MPSP* gene sequences of 15 *T. orientalis* haplotypes. The color scale represents the degree of genetic divergence, with red indicating low distance (high similarity) to yellow (intermediate distance/similarity) and green indicating high distance (low similarity). The distinct blocks along the diagonal highlight the clear clustering of isolates into four genotypes: Type 4, Type 5, Type 7, and Type N2.

Haplotype	Accession No.	PZ316041	PZ316040	PZ316042	PZ316043	PZ316044	PZ316045	PZ316046	PZ316047	PZ316048	PZ316049	PZ316050	PZ316051	PZ316052	PZ316054	PZ316053	Type
**1**	PZ316041	–															**4**
**2**	PZ316040	0.17151	–														**5**
**3**	PZ316042	0.16876	0.00887	–													**5**
**4**	PZ316043	0.17046	0.01035	0.00143	–												**5**
**5**	PZ316044	0.19369	0.24660	0.24860	0.24463	–											**7**
**6**	PZ316045	0.20020	0.25907	0.26334	0.25907	0.03275	–										**7**
**7**	PZ316046	0.20396	0.26066	0.26491	0.26437	0.03611	0.00719	–									**7**
**8**	PZ316047	0.19692	0.25509	0.25936	0.25883	0.02524	0.00723	0.01456	–								**7**
**9**	PZ316048	0.20730	0.25634	0.26777	0.26721	0.03537	0.00587	0.00146	0.01338	–							**7**
**10**	PZ316049	0.20396	0.25368	0.25797	0.25744	0.03611	0.00575	0.00430	0.01309	0.00293	–						**7**
**11**	PZ316050	0.19905	0.25278	0.25475	0.25423	0.00578	0.03308	0.03609	0.02523	0.03535	0.03609	–					**7**
**12**	PZ316051	0.19969	0.25368	0.25797	0.25744	0.03304	0.00287	0.00430	0.01016	0.00293	0.00286	0.03302	–				**7**
**13**	PZ316052	0.20261	0.25066	0.25265	0.25213	0.00873	0.03310	0.03611	0.02839	0.03537	0.03611	0.00578	0.03304	–			**7**
**14**	PZ316054	0.17378	0.25907	0.25686	0.25863	0.22133	0.22475	0.22192	0.22523	0.23105	0.22415	0.22430	0.22415	0.22133	–		**N2**
**15**	PZ316053	0.17926	0.26464	0.26236	0.26411	0.22262	0.23804	0.23292	0.23246	0.24242	0.23739	0.22556	0.23739	0.22852	0.01924	–	**N2**

**Table 5:** Mean pairwise genetic distances within (diagonal) and between (off-diagonal) *T. orientalis MPSP* genotypes. The background colors represent the degree of genetic distance, ranging from red (low distance/high similarity) to yellow (intermediate distance/similarity) and green (high distance/low similarity).

Genotype	Type 4	Type 5	Type 7	Type N2
Type 4	n/c			
Type 5	0.170	0.01		
Type 7	0.201	0.257	0.02	
Type N2	0.177	0.261	0.229	0.02

## Data Availability

Representative sequences generated in this study were deposited in the GenBank database under accession numbers PZ316040–PZ316054.

## References

[1] Herrero M., Grace D., Njuki J., Johnson N., Enahoro D., Silvestri S., Rufino M.C. (2013). The Roles of Livestock in Developing Countries. Animal.

[2] Chiariotti A., Borghese A., Boselli C., Barile V.L. (2025). Water Buffalo’s Adaptability to Different Environments and Farming Systems: A Review. Animals.

[3] El-Alfy E.S., Abbas I., Elseadawy R., Saleh S., Elmishmishy B., El-Sayed S.A.E.S., Rizk M.A. (2023). Global Prevalence and Species Diversity of Tick-Borne Pathogens in Buffaloes Worldwide: A Systematic Review and Meta-Analysis. Parasites Vectors.

[4] Sansamur C., Boonchuay K., Ngasaman R., Olana K.O.A., Punyapornwithaya V. (2025). Epidemiology and factors associated with the infection of *Babesia bigemina*, *Babesia bovis*, and *Theileria orientalis* in Thale Noi Wetland buffaloes (*Bubalus bubalis*), Southern Thailand. BMC Vet. Res..

[5] Terkawi M.A., Huyen N.X., Shinuo C., Inpankaew T., Maklon K., Aboulaila M., Igarashi I. (2011). Molecular and serological prevalence of *Babesia bovis* and *Babesia bigemina* in water buffaloes in the northeast region of Thailand. Vet. Parasitol..

[6] Altangerel K., Sivakumar T., Inpankaew T., Jittapalapong S., Terkawi M.A., Ueno A., Yokoyama N. (2011). Molecular Prevalence of Different Genotypes of *Theileria orientalis* Detected from Cattle and Water Buffaloes in Thailand. J. Parasitol..

[7] Saetiew N., Simking P., Inpankaew T., Wongpanit K., Kamyingkird K., Wongnakphet S., Jittapalapong S. (2015). Prevalence and Genetic Diversity of *Anaplasma marginale* Infections in Water Buffaloes in Northeast Thailand. J. Trop. Med. Parasitol..

[8] Nguyen A.H., Tiawsirisup S., Kaewthamasorn M. (2020). Molecular detection and genetic characterization of *Anaplasma marginale* and *Anaplasma platys*-like (*Rickettsiales: Anaplasmataceae*) in water buffalo from eight provinces of Thailand. BMC Vet. Res..

[9] Bock R., Jackson L., De Vos A., Jorgensen W. (2004). Babesiosis of Cattle. Parasitology.

[10] Watts J.G., Playford M.C., Hickey K.L. (2016). *Theileria orientalis*: A Review. N. Z. Vet. J..

[11] Homer M.J., Aguilar-Delfin I., Telford S.R., Krause P.J., Persing D.H. (2000). Babesiosis. Clin. Microbiol. Rev..

[12] Chaussepied M., Langsley G. (2018). *Theileria*-transformed bovine leukocytes have cancer hallmarks. Trends Parasitol..

[13] Kocan K.M., de la Fuente J., Blouin E.F., Coetzee J.F. (2010). The Natural History of *Anaplasma marginale*. Vet. Parasitol..

[14] Aubry P., Geale D.W. (2011). A Review of Bovine Anaplasmosis. Transbound. Emerg. Dis..

[15] AbouLaila M., Yokoyama N., Igarashi I. (2010). Development and Evaluation of a Nested PCR Based on Spherical Body Protein 2 Gene for the Diagnosis of *Babesia bovis* Infection. Vet. Parasitol..

[16] Etiang P., Kamusiime M., Wamala H., Nkamwesiga J., Ainebyoona S., Abizera H., Muhanguzi D. (2025). Prevalence and Seasonal Variation of Tick-Borne Haemoparasites in Cattle from North-Eastern Uganda. Sci. Rep..

[17] Jeong W., Yoon S.H., An D.J., Cho S.H., Lee K.K., Kim J.Y. (2010). A Molecular Phylogeny of the Benign *Theileria* Parasites Based on Major Piroplasm Surface Protein (MPSP) Gene Sequences. Parasitology.

[18] Kamani J., Schaer J., Umar A.G., Pilarshimwi J.Y., Bukar L., González-Miguel J., Harrus S. (2022). Molecular Detection and Genetic Characterization of *Anaplasma marginale* and *Anaplasma platys* in Cattle in Nigeria. Ticks Tick-Borne Dis..

[19] Masatani T., Hayashi K., Andoh M., Tateno M., Endo Y., Asada M., Kusakisako K., Tanaka T., Gokuden M., Hozumi N. (2017). Detection and molecular characterization of *Babesia*, *Theileria*, and *Hepatozoon* species in hard ticks collected from Kagoshima, the southern region in Japan. Ticks Tick-Borne Dis..

[20] Kledmanee K., Suwanpakdee S., Krajangwong S., Chatsiriwech J., Suksai P., Suwannachat P., Chaichoun K. (2009). Development of Multiplex PCR for Detection of *Ehrlichia canis*, *Babesia* spp. and *Hepatozoon canis* in Canine Blood. Southeast Asian J. Trop. Med. Public Health.

[21] Petrigh R., Ruybal P., Thompson C., Neumann R., Moretta R., Wilkowsky S., Farber M. (2008). Improved Molecular Tools for Detection of *Babesia bigemina*. Ann. N. Y. Acad. Sci..

[22] Cao S., Aboge G.O., Terkawi M.A., Yu L., Kamyingkird K., Luo Y., Xuan X. (2012). Molecular Detection and Identification of *Babesia bovis* and *Babesia bigemina* in Cattle in Northern Thailand. Parasitol. Res..

[23] Ota N., Mizuno D., Kuboki N., Igarashi I., Nakamura Y., Yamashina H., Yokoyama N. (2009). Epidemiological Survey of *Theileria orientalis* Infection in Grazing Cattle in the Eastern Part of Hokkaido, Japan. J. Vet. Med. Sci..

[24] Parola P., Roux V., Camicas J.L., Baradji I., Brouqui P., Raoult D. (2000). Detection of Ehrlichiae in African Ticks by PCR. Trans. R. Soc. Trop. Med. Hyg..

[25] de la Fuente J., Van Den Bussche R.A., Garcia-Garcia J.C., Rodríguez S.D., García M.A., Guglielmone A.A., Kocan K.M. (2002). Phylogeography of New World Isolates of *Anaplasma marginale* Based on Major Surface Protein Sequences. Vet. Microbiol..

[26] Barlough J.E., Madigan J.E., DeRock E., Bigornia L. (1996). Nested Polymerase Chain Reaction for Detection of *Ehrlichia equi* Genomic DNA in Horses and Ticks (*Ixodes pacificus*). Vet. Parasitol..

[27] Kawahara M., Rikihisa Y., Lin Q., Isogai E., Tahara K., Itagaki A., Hiramisu Y., Tajima T. (2006). Novel Genetic Variants of *Anaplasma phagocytophilum*, *Anaplasma bovis*, *Anaplasma centrale*, and a Novel *Ehrlichia* sp. in Wild Deer and Ticks on Two Major Islands in Japan. Appl. Environ. Microbiol..

[28] Seerintra T., Saraphol B., Thanchomnang T., Piratae S. (2023). Molecular prevalence of *Anaplasma* spp. in cattle and assessment of associated risk factors in Northeast Thailand. Vet. World.

[29] Seerintra T., Krinsoongnern W., Thanchomnang T., Piratae S. (2024). Molecular occurrence and genetic identification of *Babesia* spp. and *Theileria* spp. in naturally infected cattle from Thailand. Parasitol. Res..

[30] Hall T.A. (1999). BioEdit: A User-Friendly Biological Sequence Alignment Editor and Analysis Program for Windows 95/98/NT. Nucleic Acids Symp. Ser..

[31] Rozas J., Ferrer-Mata A., Sánchez-DelBarrio J.C., Guirao-Rico S., Librado P., Ramos-Onsins S.E., Sánchez-Gracia A. (2017). DnaSP 6: DNA Sequence Polymorphism Analysis of Large Data Sets. Mol. Biol. Evol..

[32] Kumar S., Stecher G., Li M., Knyaz C., Tamura K. (2018). MEGA X: Molecular Evolutionary Genetics Analysis across Computing Platforms. Mol. Biol. Evol..

[33] Lakew B.T., Eastwood S., Walkden-Brown S.W. (2023). Epidemiology and Transmission of *Theileria orientalis* in Australasia. Pathogens.

[34] Jaybhaye A., Kundu K., Jadhav Y., Pawar P. (2019). Therapeutic Management of Concomitant *Theileria orientalis* and *Babesia bovis* Infection in a Crossbred Jersey Cow. J. Vet. Parasitol..

[35] Nguyen A.H., Tiawsirisup S., Kaewthamasorn M. (2020). Low Level of Genetic Diversity and High Occurrence of Vector-Borne Protozoa in Water Buffaloes in Thailand Based on 18S Ribosomal RNA and Mitochondrial Cytochrome b Genes. Infect. Genet. Evol..

[36] Srionrod N., Nooroong P., Poolsawat N., Minsakorn S., Watthanadirek A., Junsiri W., Sangchuai S., Chawengkirttikul R., Anuracpreeda P. (2022). Molecular Characterization and Genetic Diversity of *Babesia bovis* and *Babesia bigemina* of Cattle in Thailand. Front. Cell. Infect. Microbiol..

[37] Junsiri W., Watthanadirek A., Poolsawat N., Kaewmongkol S., Jittapalapong S., Chawengkirttikul R., Anuracpreeda P. (2020). Molecular Detection and Genetic Diversity of *Anaplasma marginale* Based on the Major Surface Protein Genes in Thailand. Acta Trop..

[38] O’Neill X., White A., Gortázar C., Ruiz-Fons F. (2023). The Impact of Host Abundance on the Epidemiology of Tick-Borne Infection. Bull. Math. Biol..

[39] Thinnabut K., Rodpai R., Sanpool O., Maleewong W., Tangkawanit U. (2024). Detection of *Theileria* in Cattle Ticks (*Rhipicephalus microplus*) (Canestrini, 1888) in Upper-Northeastern Thailand. Acta Trop..

[40] Lakew B.T., Kheravii S.K., Wu S.B., Eastwood S., Andrew N.R., Nicholas A.H., Walkden-Brown S.W. (2021). Detection and Distribution of Haematophagous Flies and Lice on Cattle Farms and Potential Role in the Transmission of *Theileria orientalis*. Vet. Parasitol..

[41] El-Alfy E.S., Elseadawy R., Saleh S., Elmishmishy B., Al-Kappany Y., Abbas I. (2025). Genetic Diversity and Phylogeography of Global *Theileria orientalis* Isolates Inferred from MPSP Gene Sequences. Parasitol. Int..

[42] Subhasinghe J., Mahakapuge T.A.N., Madusanka K.S., Rajakaruna R.S., Jabbar A., Perera P.K. (2024). First report of oriental theileriosis in the Intermediate Zone, Sri Lanka: Is *Theileria orientalis type 7* always apathogenic?. Vet. Parasitol. Reg. Stud. Rep..

